# Oculocerebrocutaneous Syndrome (Delleman Syndrome): A Case with a Novel Presentation of Orbital Involvement

**DOI:** 10.1155/2021/5524131

**Published:** 2021-08-02

**Authors:** Mahbobeh Bahmani, Razieh Naseri, Alireza Iraniparast, Raya Mokhtari, Seyed Hamed Jafari

**Affiliations:** ^1^Shiraz University of Medical Sciences, Shiraz, Iran; ^2^Medical Imaging Research Center, Shiraz University of Medical Sciences, Shiraz, Iran

## Abstract

Oculocerebrocutaneous syndrome (OCCS), also known as Delleman syndrome (DS), is a rare congenital anomaly featuring focal skin defects, orbital anomalies, and central nervous system malformations. Diagnosis of Delleman syndrome is based on the triad of eye, central nervous system (CNS), and cutaneous defects and confirmed by magnetic resonance imaging. A 23-day-old girl was referred to our department for brain imaging. The infant had multiple cutaneous appendages on the right side of her face. There also was a fleshy mass measuring about 12 mm over her right eye. Brain MRI demonstrated the evidence of colpocephaly, agenesis of the corpus callosum, nodular subependymal heterotopias adjacent to the right lateral ventricle, aplasia of the cerebellar vermis, hypoplasia of the right cerebellar hemisphere, and widening of CSF space in the posterior fossa. There was also an exophytic skin lesion on her right cheek, measuring about 13 × 12 mm in size. In the orbital MRI, there was a mixed cystic solid mass measuring about 25 × 20 mm in her right orbital cavity. The orbital content was abnormal and suggestive of rudimentary orbit. Considering the findings, diagnosis of oculocerebrocutaneous syndrome (Delleman syndrome) was established for the patient. Because of the variations in orbital and CNS manifestations, all patients with clinical suspicion of DS should be assessed by brain and orbital MRI and managed by a pediatric neurologist and ophthalmologist.

## 1. Introduction

Oculocerebrocutaneous syndrome (OCCS), also known as Delleman syndrome (DS), is a rare congenital anomaly featuring focal skin defects, some form of orbital anomaly (microphthalmia/anophthalmia and orbital cysts) and central nervous system malformation (intracranial cyst). This syndrome was described first in two Dutch children by Delleman and his colleagues in 1981, and since then, about 45 cases have been reported. Some of the newly reported cases are associated with vertebral abnormalities, hydrocephalus, cleft lip, cleft palate, and facial cleft [1].

Diagnosis of Delleman syndrome is based on the triad of eye, central nervous system (CNS), and cutaneous defects and confirmed by magnetic resonance imaging (MRI) which usually demonstrates multiple ocular, cerebral, and cerebellar cysts and malformations [2].

We recently studied a patient with all the clinical and radiological features of DS.

## 2. Case Presentation

**Figure 1:**
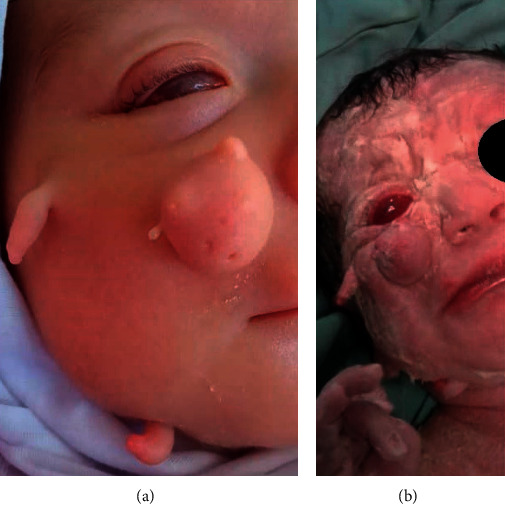
Multiple cutaneous appendages on the right side of the face and a fleshy mass under the right eye.

A 23-day-old girl was referred to our department for brain imaging. She was the second child of a nonconsanguineous couple and born at full term by caesarian section. Birth weight was 2400 g (<3rd percentile), length was 47 cm (15th percentile), head circumference was 32 cm (3rd percentile), and her Apgar score was 8/10 at one minute and 10/10 at 5 minutes. The infant had multiple cutaneous finger-like appendages on the right side of her face. There also was a fleshy mobile, skin-colored mass measuring about 12 mm over her right cheek (Figure 1).

Her sister was about 5 years old without any anomalies or developmental delay. The parents had no medical problems, including no history of abortion or miscarriage. During the pregnancy, the mother was properly under midwifery-led care and there were no antenatal or maternal problems. Also, the mother denied any teratogenic exposure during pregnancy. Physical examination revealed three finger-like, firmed, and skin-colored cutaneous appendages on her right cheek and the right periauricular and mandibular area. Ophthalmologic examination showed a fleshy mass in the place of the right orbit. The right eye was completely blind, and the left eye seemed to have a normal vision. Her echocardiogram showed no structural abnormalities. Her abdominopelvic ultrasound study was unremarkable. The extremities and genitalia were normal. Also, no report of seizure had been documented since birth.

**Figure 2:**
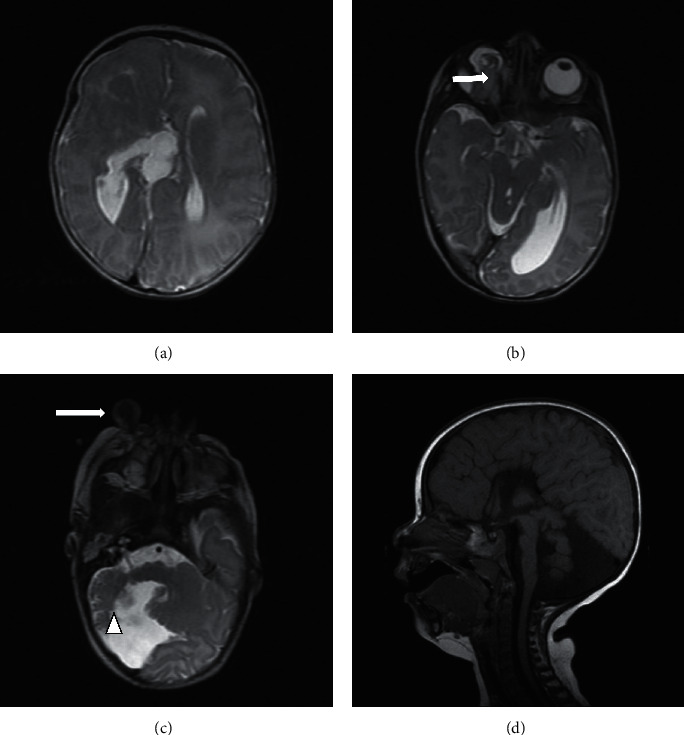
Axial images of T2W MRI demonstrated (a) colpocephaly and agenesis of the corpus callosum, (b) a mixed cystic solid structure in the right orbital cavity (black arrow), and (c) a cutaneous appendage in the right infraorbital area (white arrow), aplasia of the vermis, hypoplasia of the right cerebellar hemisphere (head arrow), and widening of the CSF space in the posterior fossa. Sagittal image of T1W MRI in midline showed (d) agenesis of the corpus callosum and cerebellar hypoplasia.

The patient was referred to the department of radiology for imaging work-up. Brain and orbit MRI was performed which showed colpocephaly and agenesis of the corpus callosum. Also, there were nodular subependymal heterotopias, adjacent to the right lateral ventricle. Aplasia of the vermis, hypoplasia of the right cerebellar hemisphere, and widening of the CSF space in the posterior fossa were some other findings in her brain MRI. There were also some skin lesions with exophytic appearance in the face up to 13 × 12 mm in the infraorbital area. In the orbital MRI, there was a mixed cystic solid mass measuring about 25 × 20 mm in her right orbital cavity. The orbital content was abnormal and suggestive of rudimentary orbit. The left orbit was completely normal (Figure 2).

Finally, based on the findings, diagnosis of oculocerebrocutaneous syndrome (Delleman syndrome) was established for the patient.

## 3. Discussion

As described before, oculocerebrocutaneous (Delleman) syndrome (OCCS or DS) is a sporadic genetic abnormality characterized by eye, CNS, and cutaneous anomalies [1].

The exact etiological mechanism of DS is still unknown, but one strong hypothesis suggests a sporadic mutation in the fifth or sixth week of development as the underlying cause in DS. This mutation may be associated with a somatic mosaicism that plays a pivotal role in ectodermal development. Another hypothesis has suggested the possibility of an association between the X chromosome and DS, resulting in the higher prevalence of this syndrome in the male population [3].

All previously reported cases seem to be sporadic, and there have been no cases of familial DS. Also, there have been no reports of its recurrence in siblings or children of affected adults in previous cases. In our case, similarly, no evidence of genetic relation and consanguinity was found [1].

To date, there are few reported cases of this syndrome. Eye anomalies in such cases have included orbital cysts, microphthalmia, anophthalmia, and palpebral colobomas [1, 2]. The current case differs clinically from the previous cases regarding the morphology of the eye anomaly. Our case had rudimentary eye, which had been described in few cases [4].

A recent case of DS by De Cock and Merizian presented with eye coloboma and a large translucent cyst protruded from the left orbit. In contrast to our case, the parents of the aforementioned case were first cousins [5].

Common cutaneous malformations in this syndrome are focal hypoplasia and aplasia. Skin tags and unusual skin appendage in the periorbital area is the characteristic of this syndrome [1]. The prevalence of focal dermal hypoplasia, punch-like defects, orbital cysts, and eyelid colobomas is estimated to be 80%, 50%, 70%, and 50% in previous cases of DS, respectively. Also, all previous cases had skin appendages on their faces [6]. Similarly, our patient showed this typical feature of DS.

CNS involvement usually includes malformations of the cortical, subcortical, and brainstem-cerebellar complex that are easily recognized in modern imagings [7]. Malformation of the midline, corpus callosum agenesis, and ventricular malformations are pathognomonic for DS and are usually seen in brain MRI, as we saw in our case. This sign supports the hypothesis that this anomaly could occur in the early stages of development, when the isthmus organizing center sets up limits between the mesencephalon and cerebellum/hindbrain [3]. This patient also showed colpocephaly, agenesis of the corpus callosum, and nodular subependymal heterotopias. Aplasia of the vermis, hypoplasia of the right cerebellar hemisphere, and widening of the CSF space in the posterior fossa were also found in the brain MRI of our patient. The distribution of brain anomalies is reported meticulously by Moog et al., and the striking asymmetry is a universal aspect of brain involvement while the left side is affected most of the times the frontal lobe is more often involved [7, 8]. The MRI of our patient revealed hypoplasia of the right cerebellar hemisphere and widening of the CSF space in the posterior fossa.

Similarly, a DS case reported by Ortiz-Basso et al. had bilateral aplasia cutis and slight hypoplasia of the central vermis. However, he also had a small lesion of aplasia cutis in the lumbar area surrounded by hair. That case had several pits on the cheek [3]. Our case too had some skin pits on the skin appendage of her right cheek.

In some previous cases, palatine fissure, lip cleft, and facial cleft were seen too [7].

The other less-frequent anomalies in previously reported cases with DS are urogenital anomalies, rib defects, and ear and nose malformations [1]. But, our case didn't have these features.

In conclusion, because of the variations in orbital and CNS manifestations, all patients with clinical suspicion of DS should be assessed by brain and orbital MRI and managed by a pediatric neurologist and ophthalmologist.

## References

[1] Hunter A. G. W. (2006). Oculocerebrocutaneous and encephalocraniocutaneous lipomatosis syndromes: blind men and an elephant or separate syndromes?. *American Journal of Medical Genetics Part A*.

[2] Álvarez-Barazarte A. (2018). Delleman Oorthuys syndrome, triad of oculo-cerebral-cutaneous malformations. *Salud Arte Y Cuidado*.

[3] Ortiz-Basso T., Vigo R., Iacouzzi S., Prémoli J. (2014). Delleman (oculocerebrocutaneous) syndrome: case report. *Indian Journal of Ophthalmology*.

[4] Badejo O. A., Fasina O., Balogun J. A., Ogunbiyi J. O., Shokunbi M. T. (2018). Delleman–Oorthuys syndrome (oculocerebrocutaneous syndrome) in a Nigerian child: a case report. *Therapeutic Advances in Ophthalmology*.

[5] De Cock R., Merizian A. (1992). Delleman syndrome: a case report and review. *British Journal of Ophthalmology*.

[6] Pascual-Castroviejo I., Pascual-Pascual S. I., Velazquez-Fragua R., Lapunzina P. (2005). Oculocerebrocutaneous (Delleman) syndrome: report of two cases. *Neuropediatrics*.

[7] Moog U., Dobyn W. B. (2018). An update on oculocerebrocutaneous (Delleman-Oorthuys) syndrome. *American Journal of Medical Genetics Part C: Seminars in Medical Genetics*.

[8] Moog U. (2009). Encephalocraniocutaneous lipomatosis. *Journal of Medical Genetics*.

